# Thermal Degradation of Polymer Composites Based on Unsaturated-Polyester-Resin- and Vinyl-Ester-Resin- Filled Kraft Lignin

**DOI:** 10.3390/ma18030524

**Published:** 2025-01-23

**Authors:** Nadiia V. Siharova, Przemysław Pączkowski, Yuriy I. Sementsov, Serhiy V. Zhuravsky, Mykola V. Borysenko, Andriy D. Terets, Olexandr V. Mischanchuk, Mariia I. Terets, Yulia V. Hrebelna, Barbara Gawdzik

**Affiliations:** 1 Chuiko Institute of Surface Chemistry, NAS of Ukraine, 17 General Naumov Str., 03164 Kyiv, Ukraine; ysementsov@ukr.net (Y.I.S.); s_zhur@ukr.net (S.V.Z.); borysenko@yahoo.com (M.V.B.); terets.andrey@gmail.com (A.D.T.); bigsnake@i.ua (O.V.M.); teretsmariya@gmail.com (M.I.T.); liza50@ukr.net (Y.V.H.); 2 Department of Biotechnical Problems of Diagnostics IPCC, NAS of Ukraine, 42/1 Nauky Prosp., 03028 Kyiv, Ukraine; 3 Department of Polymer Chemistry, Institute of Chemical Sciences, Faculty of Chemistry, Maria Curie-Sklodowska University, Gliniana 33, 20-614 Lublin, Poland; przemyslaw.paczkowski@umcs.pl (P.P.); barbara.gawdzik@umcs.pl (B.G.); 4 Ningbo Sino-Ukrainian New Materials Industrial Technologies Institute, Kechuang Building, N777 Zhongguan Road, Ningbo 315211, China; 5 Taras Shevchenko National University of Kyiv, 60 Volodymyrska Street, 01033 Kyiv, Ukraine

**Keywords:** unsaturated polyester resin, vinyl ester resin, kraft lignin, thermophysical properties, thermal degradation, activation energy, polymer structure

## Abstract

The creation of heat-resistant polymers represents one of the most significant challenges and priorities in contemporary scientific research. The incorporation of a filler of analogous nature and content into disparate types of resins will facilitate the identification of the relationship between properties and the structure of macromolecular chains in synthetic resins that function as composite matrices. The objective of this study was to ascertain the impact of lignin at 5 and 15% by weight on the thermal degradation of two resin-based composites with disparate structural compositions. The thermal decomposition products of the composites were determined by the method of temperature-programmed desorption mass spectroscopy (TPD MS). The thermal oxidative degradation patterns of polymer composites were investigated through derivatography (Q–1500D). It was demonstrated that the incorporation of lignin in modest quantities has a negligible impact on the thermal stability of the composites. Notably, the temperature at which the composites undergo thermal decomposition during thermal oxidation degradation exhibits a variation of over 10 °C, suggesting that the utilisation of lignin holds promise for the development of environmentally benign and cost-effective materials for diverse industrial applications.

## 1. Introduction

The increasing demand for high-strength, environmentally friendly, and durable materials is driving the development of unsaturated polyester (UPR) and vinyl ester (VER) resin production technologies. In this context, research is focused on enhancing the properties of resins, reducing their adverse environmental impact and optimising production costs. Unsaturated polyester and vinyl ester resins represent the primary thermosetting polymers employed in the manufacture of composite materials. Despite their similarities, these resins exhibit key differences that determine their characteristics and applications. UPR and VER are extensively employed in the manufacture of composite materials, coatings, and adhesives, largely due to their robust mechanical properties, chemical resistance, and capacity to cure at low temperatures [1,2].

Unsaturated polyester resins with a reduced styrene content are regarded as environmentally friendly materials with considerable potential for utilisation in green technologies [3].

A substantial body of research has demonstrated the practical applications of lignin in polyester resins, including its ability to enhance the strength and environmental sustainability of composites. In particular, the works cited in references [4,5,6,7,8] describe studies on the practical use of synthetic polyester resins filled with lignin and kraft lignin.

Lignin, the biomaterial of organic origin and an irregular polymer comprising branched macromolecules, is the subject of intensive research due to its capacity to undergo a multitude of chemical transformations as a consequence of the extensive number of active functional groups [9,10]. Lignin, with varying structures and properties, can be derived from diverse sources, including trees, crops, and other plants [11], through biochemical, physical, or chemical processing [12].

Lignin can be divided into two main categories, distinguished by the method of extraction: sulphur-containing and sulphur-free lignin [13]. Sulphur-containing lignin is derived from cellulose, as in the paper and pulp industry, and includes kraft lignin and lignosulfonates. Lignin that does not contain sulphur, such as soda or alkaline lignin and organosoluble lignin, is produced, for example, in the context of bioethanol production processes [14,15].

Kraft lignin is distinguished by a highly condensed structure comprising single-C bonds, along with a substantial number of phenolic hydroxyl groups, which are the result of β-aryl bond cleavage [16]. It can be dissolved in water under specific conditions [17]. Lignosulfonates, conversely, are water-soluble and contain a substantial quantity of sulphur in the form of SO_3_^−^ and HSO_3_^−^ groups [18].

Lignin, a biocompatible and renewable polymer, can be employed as a supplement to photocatalysts in photovoltaic cells, thereby enhancing the efficiency of solar cells and electronic devices [19]. Lignin can also be employed in the creation of diverse adhesives and coatings [20]. In particular, lignin has great potential in medicine due to its antioxidant, antibacterial, and biocompatible properties. Its use is becoming increasingly popular in the development of innovative therapeutic and diagnostic materials. Lignin nanoparticles serve as effective carriers for targeted drug delivery, especially in cancer treatment. Lignin materials are highly biocompatible, making them safe for medical use.

The utilisation of lignin, including its preparation and modification, represents a significant area of opportunity for the development of innovative materials [21]. For instance, lignin with COOH, OH, and OCH_3_ functional groups is a promising candidate for polymers such as polyurethanes, epoxies, and polyester resins [22].

The synthesis and utilisation of lignin and polyesters in green chemistry for the creation of biodegradable composites have been the subject of investigation [4], as have modifications to lignin for polyester resins with a view to enhancing their mechanical properties [5]. Furthermore, lignin is employed to enhance the heat resistance of polyester resins [8]. Polyester resins filled with kraft lignin are employed in a range of industrial applications, including the production of automotive and construction materials and corrosion protection surfaces [6,23]. The utilisation of lignin in polyester resins has been the subject of investigation with a view to reducing costs and enhancing environmental performance. Zaghloul et al. report that the addition of filler leads to a reduction in the cost of polyester resin composites [24]. Studies of Hodásová et al. show that lignin can be used as an alternative to polyacrylonitrile (PAN) in the production of carbon fibres, which can reduce their cost from 18–26 USD kg^−1^ to 7–11 USD kg^−1^ [25].

The effect of lignin and modified lignin on the mechanical properties of the ternary composite system styrene/unsaturated polyester/lignin, as well as in polypropylene composites, has already been studied by researchers [26,27]. The use of lignin and its production and modification open up significant opportunities for the creation of new materials.

Numerous publications address the practical utilisation of vinyl ester resins incorporating lignin or Kraft lignin. The study in [28] examines the utilisation of Lignoboost lignin in bio-based vinyl ester resins, with a particular focus on the enhancement of mechanical properties through the incorporation of lignin. The dissertation in [29] examines the potential of using lignin as a substitute for petroleum-based reactive diluents in vinyl ester resins, with the aim of enhancing their environmental sustainability and mechanical properties. The patent in [30] presents a method for the production of vinyl-ester-based composites utilising lignin as a filler, with the objective of enhancing material performance and reducing environmental impact. The study in [31] emphasises the influence of the addition of lignin. The addition of lignin to vinyl ester resins has been demonstrated to enhance both the mechanical and thermal properties of the resulting material, rendering it suitable for the production of high-performance composites. The paper in [32] outlines methodologies for the fabrication of fusible lignin composites, which employ lignin in diverse forms (e.g., the H-form of lignin) in conjunction with reactive molecules to augment the mechanical and thermal properties of a polyester resin.

The porous structures of lignin materials are used to create biocompatible platforms for tissue regeneration. Due to their high mechanical strength and biodegradability, these materials are used in surgery and dentistry. Lignin derivatives exhibit a significant antimicrobial effect, which can be useful in the development of medical dressings and protective coatings. For example, work [33] focuses on the antioxidant properties of lignin and its use in drug delivery. An overview of lignin porous biomaterials for drug delivery control, tissue engineering, wound dressing, and medical sensors, as well as the use of lignin nanoparticles for drug delivery and their role in the development of anticancer and antibacterial agents, is presented [34,35].

The majority of global research is focused on the utilisation of polymer composites incorporating kraft lignin as a filler material, with the objective of enhancing the structural integrity and durability of polyester resins in composite materials. The research encompasses the utilisation of lignin-filled polyester resins in coatings, with a particular emphasis on enhanced resistance to ultraviolet radiation and environmental wear and tear. Inventions in this field elucidate a methodology for the improvement of resins with kraft lignin, with the objective of enhancing their adhesion and mechanical properties for utilisation in the production of adhesives and coatings. This includes the creation of bio-based polyester composites, where lignin serves as a filler, thereby improving the stability and performance of the resin for use in building materials.

## 2. Materials and Methods

### 2.1. Materials

In this work, composites based on two types of resins were investigated: 1—a mixture of unsaturated orthophthalic polyester resin (UPR) based on recycled PET (Polyethyleneterephthalate) with styrene, Estromal 14PB-06 NZ manufactured by LERG (Pustków, Poland) and 2—vinyl ester/styrene resin (VER) EBE1 (Pustków, Poland).

**Figure 1:**
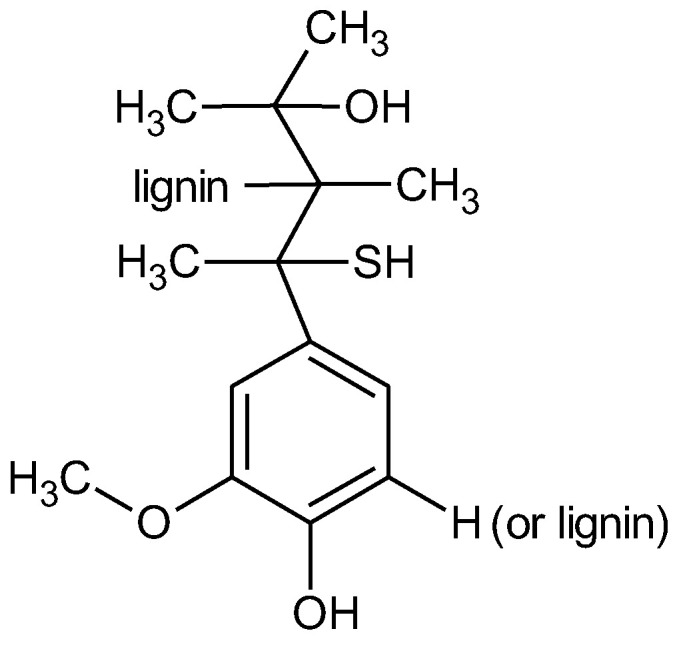
Structural formula of kraft lignin.

Methyl ethyl ketone peroxide (MEKP, Luperox DHD-9) (St. Louis, MO, USA) was used as a reaction initiator. A 4% solution of polymeric cobalt [13] was used as a reaction accelerator, which was synthesised at the Department of Polymer Chemistry, Institute of Chemical Sciences, Maria Curie-Skłodowska University (Lublin, Poland). Kraft lignin was used as a filler, and lye with a low sulfonate content was purchased from Sigma-Aldrich (St. Louis, MO, USA). A generalised formula given on the manufacturer’s website with characteristic groups and bonds is shown in Figure 1.

The composites were prepared by mixing until homogeneous with a mechanical stirrer. The composites consisted of UPR or VER, to which 5 or 15 wt% of kraft lignin, 1.1 wt% Luperox DHD-9, and 0.25 wt% of a 4% cobalt solution were added, and the use of DEA (*N*,*N*′-diethylaniline) as a co-accelerator was 0.06 wt%. The materials were cured at room temperature for 24 h and then heated at 80 °C for 10 h for further curing.

**Figure 2:**
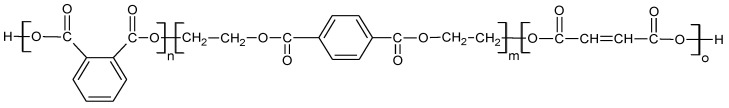
Typical structural formula of the polymer chain of unsaturated polyester.

**Figure 3:**
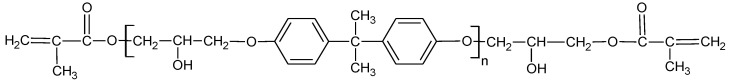
Typical structural formula of the polymer chain of vinyl ester.

Unsaturated polyester resin (UPR) contains double bonds (-C=C-) that allow it to polymerise with styrene or other monomers to form a three-dimensional mesh (Figure 2). The structural differences between these resins are the presence of double and ether bonds, as well as the structure of the main polymer chain: in unsaturated polyester resin, the chain consists of repeating units of acids and glycols, while, in vinyl ester resin (Figure 3), the main chain is derived from epoxy resin with terminal unsaturated groups. These structural differences explain the difference in thermal properties of the resins.

### 2.2. Methods

#### 2.2.1. Thermal Properties

To study the thermal properties of the composites, the temperature-programmed desorption mass spectrometry (TPD-MS) and differential scanning calorimetry (DSC) were used.

The composition of volatile products of the thermal degradation of composites was determined by the TPD-MS method using an MX 7304A mass-spectrometer (Sumy, Ukraine), with temperature control in the range from 25 to 800 °C under vacuum conditions. The mass spectra of the destruction products of the samples were recorded and analysed using an automated computer-based system. The spectra were recorded in the range of *m*/*z* 15–200 a.u.m. The desorption curves of the curvature decomposition of resin–lignin composites were obtained from the mass spectrometric analysis data.

The thermal properties of the polymer composites were studied using a Q–1500D derivatograph (MOM, Budapest, Hungary) equipped with a computerised data recording system. Samples weighing 50 mg were placed in a ceramic crucible and heated at a rate of 10 °C min^−1^ in an air atmosphere. Measurements were performed in the temperature range from room temperature to 1000 °C.

#### 2.2.2. Calculation of Activation Energy of Desorption

The activation energy E_d_ of the thermal destruction of composites was calculated using the Wigner–Polanyi and Redhead equations.

For different temperature ranges, the activation energy of desorption was determined by the generalised Redhead formula [36]:

(1) $$ln\left(\frac{T_{m}^{2}}{\beta }\right)=\frac{E_{d}}{R\cdot T_{m}}\;\to \;E_{d}=R\cdot T_{m}\cdot ln\left(\frac{T_{m}^{2}}{\beta }\right)$$

where *T_m_* is the temperature of the maximum curve, *R* is the gas constant, and *β* is the heating rate.

Wigner–Polanyi formula [37] is as follows:

(2) $$E_{d}=\frac{ln\left(\frac{\theta_{1}}{\theta_{2}}\right)\cdot R\cdot \left(T_{1}\cdot T_{2}\right)}{\left(T_{2}-T_{1}\right)}$$

where *T*_1_ and *T*_2_ are the temperature values in the middle of the maximum intensity of the desorption spectrum (from TPD MS data), *R* is the universal gas constant, *R* = 8.314 J·mol^−1^·K^−1^, and *θ*_1_ and *θ*_2_ are the areas under the curve corresponding to the relative amount of volatile products from *T*_1_ and *T*_2_, respectively.

## 3. Results and Discussion

Despite the extensive research conducted on unsaturated polyester resin over an extended period, the investigation of its characteristics when combined with organic substances remains a topic of significance [38,39,40].

The investigation of the thermal degradation of lignin-filled unsaturated polyester (UPR) and vinyl ester (VER) resin composites is crucial for elucidating the decomposition products and degradation mechanisms. This enables the assessment of the impact of lignin on the thermal stability of composites and facilitates the optimisation of their composition. The study of thermophysical properties, in particular the thermal stability and thermal decomposition processes of polymer composites, is of significant importance for determining their performance characteristics. Furthermore, it facilitates a deeper comprehension of the structural transformations of the polymer matrix reinforced with a filler of plant origin. This field of research has been the subject of numerous scientific publications [41,42,43,44,45].

Yan et al. [41] investigated the thermal decomposition of kraft lignin in an atmosphere of argon, hydrogen, and carbon dioxide using the TPD-MS method. The presence of hydrogen contributed to the cleavage of lignin side chains, thereby intensifying the formation of compounds such as methane (CH_4_), benzene (C_6_H_6_), formaldehyde (HCHO), phenol (C_6_H_5_OH), and methanol (CH_3_OH). The elemental and morphological analysis of the C-H-O-N-S solid products revealed that the lowest carbon content and highest oxygen content were observed when an argon atmosphere was employed, whereas the use of a hydrogen or CO_2_ atmosphere resulted in an increase in carbon content in the final solid products. The products obtained under CO_2_ or hydrogen atmosphere were observed to contain spherical nanoparticles.

It is expected that the results obtained will provide important information about the thermal decomposition processes of composites and will contribute to the development of environmentally friendly materials with increased thermal stability. Previous studies have shown that polyester (UPR) and vinyl ester resins (VER), as well as their composites with kraft lignin, demonstrate high resistance to destructive effects, with vinyl ester resins being more resistant [38].

The investigation of the thermal degradation of lignin-filled UPR and VER composites is a crucial aspect of the development of materials with enhanced properties. The influence of lignin on the thermal stability and environmental performance of composites depends on its content and origin, with the potential to either enhance or reduce these properties. Nevertheless, in order to guarantee the dependability and longevity of these composites, it is essential to conduct a thorough examination of their thermal stability. Further research will facilitate the optimisation of composite composition, thereby enhancing performance and facilitating the expansion of their applications. As is the case with the majority of organic polymers, polyester and vinyl ester resins are subject to thermal degradation when exposed to elevated temperatures. It is, therefore, crucial to gain an understanding of the mechanisms involved in this degradation process in order to enhance the thermal stability of resins and guarantee their effective utilisation in high-temperature applications.

The aim of this study was to evaluate the effect of kraft lignin on the thermal stability of polyester and vinyl ester resins, in particular on the processes of the thermal decomposition and thermal oxidation degradation of composites. This study determined the thermal decomposition products, the activation energy of desorption of decomposition products, and the patterns of thermal oxidative degradation of composites depending on the content of kraft lignin.

The thermal decomposition processes of the composites and the identification of the main decomposition products were carried out based on the results of TPD-MS. The structure of kraft lignin contains hydroxyl and carboxyl groups, which makes it a reactive filler for polymer composites. Thus, kraft lignin can significantly affect the thermal properties and decomposition products of polymer composites, which should be taken into account when developing materials with predictable properties.

**Figure 4:**
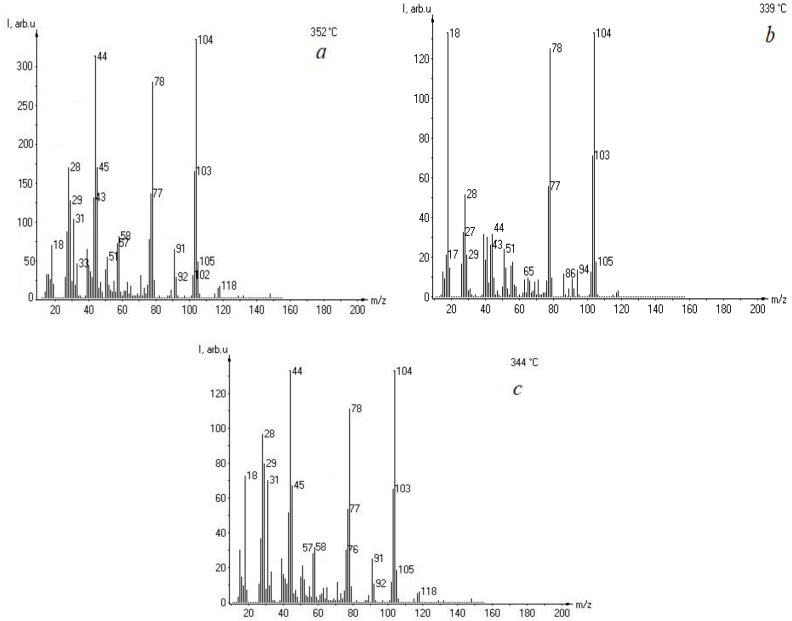
Mass spectra of unsaturated polyester resin (UPR) (**a**) and its composites with 5 wt% (**b**) and 15 wt% (**c**) of kraft lignin.

A comparison of the mass spectra of the pure polyester resin and the composite with lignin (Figure 4) revealed that the incorporation of lignin did not alter the atomic composition of the products of decomposition. The mass spectra demonstrate that the primary products of decomposition are *m*/*z* values within the interval of 120 a.u.m. Thermal decomposition results in the formation of products with *m*/*z* values of 18 (water, H_2_O), 28 (carbon monoxide, CO), 44 (carbon dioxide, CO_2_), and 78 (phenol, C_6_H_5_OH), as well as phenolic compounds with *m*/*z* values of 91 (C_7_H_5_), 94 (C_7_H_5_O), and 104 (C_6_H_5_C=CH_2_). It should be noted that lignin has the potential to facilitate the formation of phenols and their derivatives with *m*/*z* 78, 91, and 104, which have significant implications for the chemical stability of composite materials. It should be noted that there are differences in the atomic composition of the products of decomposition with *m*/*z* values between 77 and 94, which confirms the difference between the structural construction of composites of UPR and VER.

It is likely that carbon dioxide (CO_2_) and carbon monoxide (CO) are formed during the decomposition of carbon-based structures, which are components of polyester chains. CO_2_ is attributed to the decomposition of ester, carboxyl, and/or anhydride groups: acid groups in which carbon is connected to two oxygen atoms. The groups generating CO are oxygen groups, i.e., phenolic, carbonyl, and/or ether groups, e.g., weakly acidic, neutral, and basic groups, in which C is connected to one O atom.

**Figure 5:**
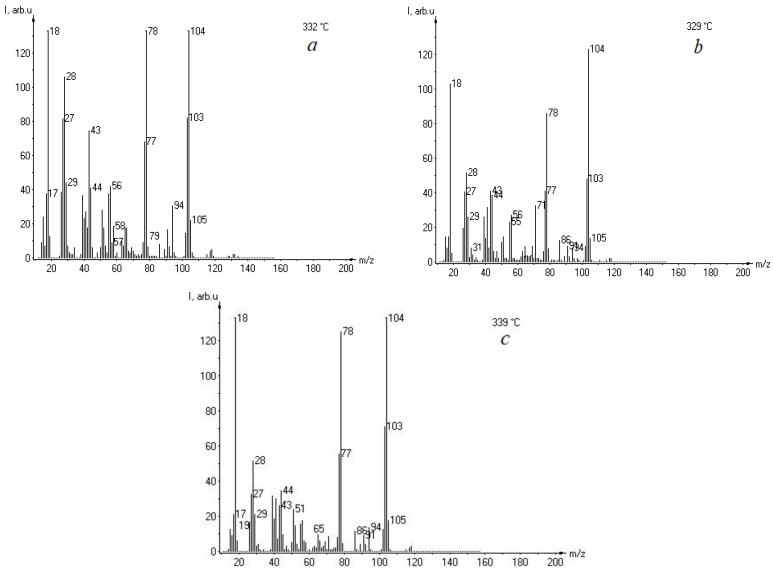
Mass spectra of vinyl ester resin (VER) (**a**) and its composites with 5 wt% (**b**) and 15 wt% (**c**) of kraft lignin.

According to the mass spectrometric analysis of the thermal decomposition of vinyl ester resin, the same volatile thermal degradation products were identified as those of polyester resin, except for phenolic compounds such as *m*/*z* 94 (C_6_H_6_O). As can be seen from Figure 5, lignin reduces the intensity of the lines in the mass spectrum, which may indicate an increase in the thermal stability of the composites.

**Figure 6:**
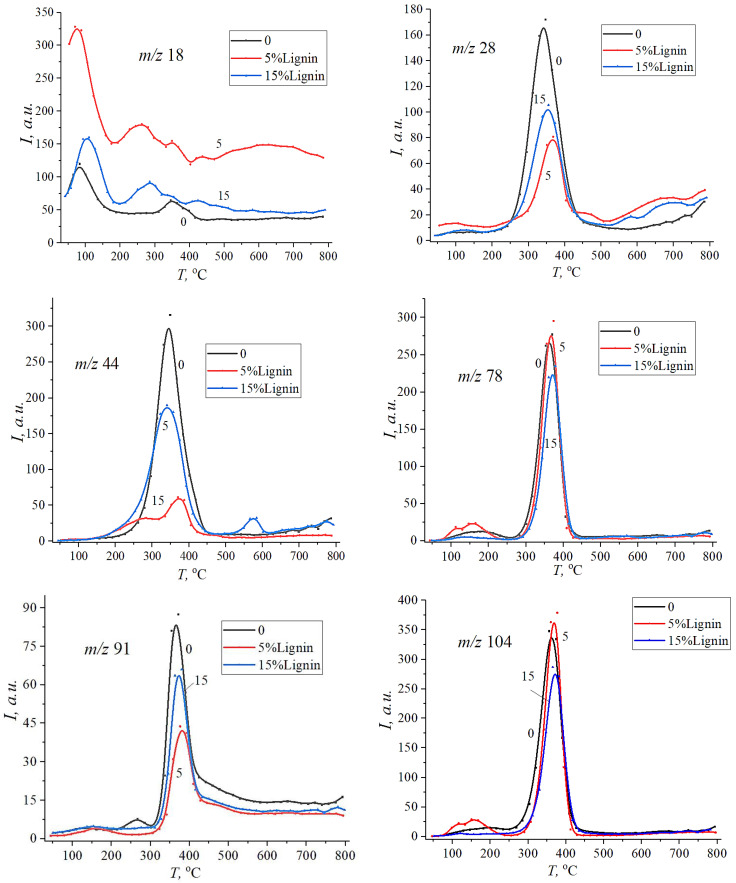
Desorption curves of decomposition fragments of pure unsaturated polyester resin (0) and its composites with 5 wt% and 15 wt% of kraft lignin.

**Figure 7:**
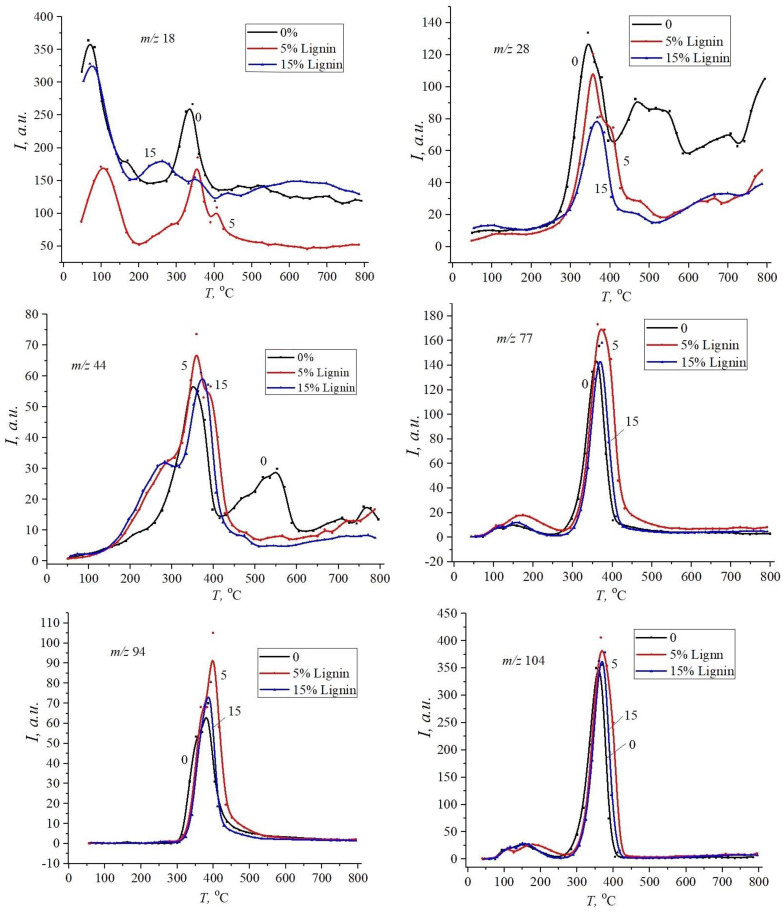
Desorption curves of decay fragments of unfilled vinyl ester resin (0) and its composites with 5 wt% and 15 wt% of kraft lignin.

Figure 6 and Figure 7 show the desorption curves reflecting the thermal decomposition of native polyester and vinyl ester resins, as well as their composites with lignin at 5 and 15 wt%. According to the data obtained, decomposition products with *m*/*z* 18 (H_2_O), 78 (C_6_H_6_) (benzene ring), and 104 (styrene) (C_6_H_5_C=CH_2_) demonstrate the first phase of degradation in the temperature range up to 250 °C. This indicates that fragments of chains that are thermally stable only up to a certain temperature are destroyed in the polymer structure of the resin and its composites with lignin.

When the temperature increases from 250 °C to 450 °C, the maximum desorption rate is observed, which corresponds to the intensive thermal decomposition of the composites. Obviously, with increasing temperature, the rate of thermal degradation increases, so it is important to determine the temperature limits within which the composite retains its properties. As can be seen from the figures, the addition of lignin to polyester resin reduces the initial decomposition temperature of the composite and increases the temperature of the maximum desorption peaks.

Lignin probably promotes the formation of carbon residues, which increases the thermal stability of the composite in certain temperature ranges. In particular, kraft lignin, as a biopolymer with a high content of aromatic rings, can improve the thermal stability of the polymer. The effect is explained by the formation of interactions between the polymer matrix and lignin due to the formation of hydrogen bonds between (-COO-) with hydroxyl and carboxyl groups of lignin in polyester resin and carbon–carbon double bonds (-C=C-) in vinyl ester resin. Lignin can contribute to the formation of localised degradation points in composites, which makes the process more intense but starts at lower temperatures. In polyester resin, lignin is evenly dispersed due to the strong adhesion between the matrix and lignin particles, which reduces local stress concentrations. In vinyl ester resin, insufficient (poor) interaction between lignin and the matrix leads to the formation of micropores and weak zones, which become centres of thermal degradation.

As can be seen from Figure 7, the addition of lignin to vinyl ester resin increases the intensity of the thermal degradation of composites, while slightly decreasing the temperature of the onset of desorption and increasing the temperature of the maximum desorption peaks. This indicates that lignin affects the mechanism of the thermal decomposition of composites. Presumably, during heating, kraft lignin decomposes to form volatile products that can interact with the polymer matrix, affecting its decomposition. It is worth noting the appearance of a second temperature peak in the range of 400–500 °C for CO and CO_2_ in the native vinyl ester resin.

At the same time, it should be noted that lignin filling does not cause significant changes in the composition of the thermal decomposition products of unsaturated polyester (UPR) and vinyl ester resin (VER), which does not affect the final composition of thermal decomposition products compared to pure polymer.

**Figure 8:**
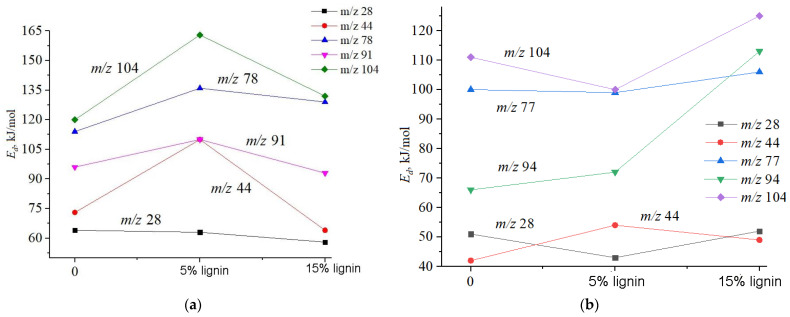
Activation energy of degradation of (**a**) unsaturated polyester resin (0) and its composites with 5 wt% and 15 wt% of kraft lignin and (**b**) vinyl ester resin (0) and its composites with 5 wt% and 15 wt% of kraft lignin.

To determine the kinetic parameters of the thermal degradation of polyester resin, vinyl ester resin, and their composites with kraft lignin, the activation energy of degradation was calculated from the TPD-MS thermograms and derivatographic curves (Table 1 and Table 2). The values of the activation energy of degradation (*E_d_*) for UPR are shown in Figure 8a and for vinyl ester resin in Figure 8b.

As can be seen in Figure 8, the introduction of 5 wt% lignin into the polymer composite increases the activation energy of degradation, while the introduction of 15 wt% lignin slightly decreases the activation energy. For vinyl-ester-based composites, the opposite trend is observed compared to unsaturated polyester resin (UPR) composites. This difference is probably due to differences in the properties of the resins themselves, which leads to different degradation mechanisms.

This difference is likely to be due to differences in the number and types of chemical bonds in the resins themselves, which leads to different degradation mechanisms. The high content of ester groups (-COO-) in polyester resin, which can form hydrogen bonds with polar groups of lignin (hydroxyl and carboxyl groups), enhances the interaction between the matrix and lignin, which contributes to the formation of a more stable composite structure. This increases the activation energy of degradation, as more energy is required to break these bonds.

Vinyl ester resin has a structure with a much smaller number of ester groups than polyester resin, a larger number of carbon–carbon double bonds (-C=C-), and lack of polar functional groups, which interact less actively with lignin, so lignin in this case does not contribute to a significant strengthening of the matrix. On the contrary, it can cause micro-defects or uneven heat distribution, which reduces the initial degradation temperature and reduces the activation energy.

The activation energy of degradation was determined using the generalised Redhead formula (Equation (1)) [34] based on thermogravimetric analysis curves. As can be seen from Table 1 and Table 2, the value of the activation energy for thermo-oxidative degradation decreases slightly over the entire range of filler concentrations: for UPR, E_d_ = 90.8–89.2 kJ·mol^−1^, and for VER, E_d_ = 108.5–102.7 kJ·mol^−1^.

The results obtained by the TPD-MS method concerning composites of unsaturated polyester resin with carbon nanotubes (CNTs) are given in [46]. It was found that the results are influenced by interactions between the polymer matrix and groups present in the filler structure. It was noticed that the course of the TPD-MS spectra depends on the CNT content in the composite. A similar tendency can be observed for the values of the destruction activation energy.

**Figure 9:**
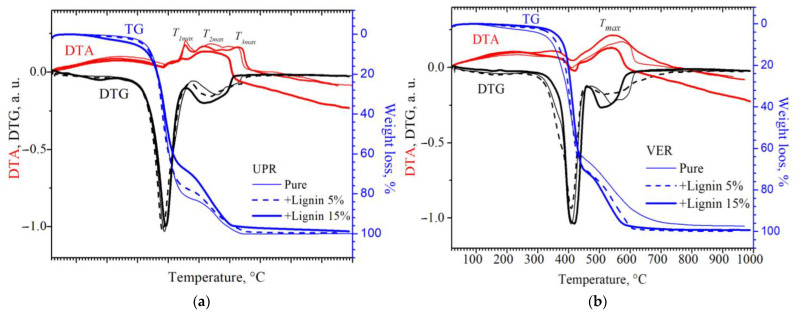
Thermogravimetric analysis (TG/DTG/DTA) of (**a**) unsaturated polyester resin and its composites with kraft lignin and (**b**) vinyl ester resin and its composites with kraft lignin.

The thermal destruction of native polyester resin and lignin composites, according to the DTG and DTA curves, occurs in three temperature intervals (Figure 9a):

(1) for the native resin with a temperature of T_max1_ = 456 °C, T_max2_ = 540 °C, and T_max3_ = 598 °C;
(2) for a resin with 5 wt% lignin with a temperature of T_max1_ = 455 °C, T_max2_ = 517 °C, and T_max3_ = 645 °C;
(3) for a resin with 15 wt% lignin with a temperature of T_max1_ = 445 °C and T_max2_ = 510 °C.

As can be seen from Figure 9a, the mass loss in the native polyester resin at T ≥ 400 °C is greater than in the lignin composites, while the opposite thermal decomposition behaviour is observed in the vinyl ester resin.

The thermal degradation of VER and composites with lignin, according to the DTG and DTA curves, is characterised by three temperature maxima (Figure 9b) for unfilled resin T_m1_ = 580 °C, for resin with 5% lignin T_m2_ = 545 °C, and for a composite with 15% lignin T_m3_ = 540 °C. As can be seen from Figure 9b, the main process of the oxidative degradation of vinyl ester resin begins at 280–300 °C and reaches a maximum at 380 °C, after which the combustion process becomes less intense due to condensed polyaromatic residues (carbonizate) in the temperature range up to 650 °C. Carbonizate occurs as a result of decomposition and reactions involving carbon chains that reorganise into more stable heat-resistant structures. The aromatic rings that make up the original polymer can cross-link or cyclise to form a stable carbon phase.

## 4. Conclusions

The addition of kraft lignin to unsaturated polyester (UPR) and vinyl (VER) resins has a minimal impact on their thermal stability and degradation products. The composition of the decomposition products remains virtually unchanged, but lignin affects the onset temperature and maximum intensity of thermal degradation.

The preservation or slight improvement of the properties of UPR composites with lignin is explained by stronger adhesive bonds formed as a result of chemical and physical interactions between the polar groups of lignin and the polar groups of polymer molecules. At the same time, lignin-filled vinyl ester composites show an increased intensity of thermal degradation and a slight decrease in the initial decomposition temperature because the polymer chains of the resin contain significantly fewer polar groups capable of forming hydrogen bonds with lignin.

Further studies of thermal decomposition at different lignin concentrations will contribute to the development of composite materials with heat-resistant properties that can be adjusted.

## Figures and Tables

**Table 1:** Activation energy for the destruction of pure unsaturated polyester resin (0) and its composites with 5 wt% and 15 wt% of kraft lignin, according to the results of DSC studies.

Sample	$E_{d_{1}}$, J·mol^−1^·K^−1^	$E_{d_{1}}$, eB	$E_{d_{2}}$, J·mol^−1^·K^−1^	$E_{d_{2}}$, eB	$E_{d_{3}}$, J·mol^−1^·K^−1^	$E_{d_{3}}$, eB
pure UPR	90.8	0.94	102.7	1.06	110.0	1.14
UPR + 5 wt% lignin	90.6	0.94	99.4	1.03	107.7	1.11
UPR + 15 wt% lignin	89.2	0.92	98.4	1.03	-	-

**Table 2:** Activation energy for the destruction of pure vinyl ester resin (0) and its composites with 5 wt% and 15 wt% of kraft lignin, according to the results of DSC studies.

Sample	$E_{d_{1}}$, J·mol^−1^·K^−1^	$E_{d_{1}}$, eB
pure VER	108.5	1.12
VER + 5 wt% lignin	103.4	1.07
VER + 15 wt% lignin	102.7	1.06

## Data Availability

The raw data supporting the conclusions of this article will be made available by the authors on request.
